# Predicting Maize Theoretical Methane Yield in Combination with Ground and UAV Remote Data Using Machine Learning

**DOI:** 10.3390/plants12091823

**Published:** 2023-04-28

**Authors:** Ardas Kavaliauskas, Renaldas Žydelis, Fabio Castaldi, Ona Auškalnienė, Virmantas Povilaitis

**Affiliations:** 1 Institute of Agriculture, Lithuanian Research Centre for Agriculture and Forestry, Instituto Ave. 1, 58344 Akademija, Lithuania; 2 Institute of BioEconomy, National Research Council of Italy (CNR), Via Giovanni Caproni 8, 50145 Firenze, Italy

**Keywords:** maize growth stages, multispectral image, phenology, remote sensing, vegetation indices

## Abstract

The accurate, timely, and non-destructive estimation of maize total-above ground biomass (TAB) and theoretical biochemical methane potential (TBMP) under different phenological stages is a substantial part of agricultural remote sensing. The assimilation of UAV and machine learning (ML) data may be successfully applied in predicting maize TAB and TBMP; however, in the Nordic-Baltic region, these technologies are not fully exploited. Therefore, in this study, during the maize growing period, we tracked unmanned aerial vehicle (UAV) based multispectral bands (blue, red, green, red edge, and infrared) at the main phenological stages. In the next step, we calculated UAV-based vegetation indices, which were combined with field measurements and different ML models, including generalized linear, random forest, as well as support vector machines. The results showed that the best ML predictions were obtained during the maize blister (R2)–Dough (R4) growth period when the prediction models managed to explain 88–95% of TAB and 88–97% TBMP variation. However, for the practical usage of farmers, the earliest suitable timing for adequate TAB and TBMP prediction in the Nordic-Baltic area is stage V7–V10. We conclude that UAV techniques in combination with ML models were successfully applied for maize TAB and TBMP estimation, but similar research should be continued for further improvements.

## 1. Introduction

The growth of the global human population, being one of the major challenges for humanity, encourages and increases energy demand at the household, smallholder, and industrial scales. Anaerobic digestion of organic materials has been developed for more than 100 years and currently has great potential in contributing to sustainable energy production [1]. During the anaerobic process, it is possible to convert fermentable biomass, including the organic fraction of municipal solid waste, food waste, green wastes, aquatic biomass, and crops [2], into a multipurpose fuel (CH_4_) and fertilizers that can be used in crop management systems while also being an effective way to reduce greenhouse gas emissions, as compared to traditional fossil fuels [3,4].

A study of digestion [5] highlighted that among the variety of substrates suitable for anaerobic digestion, agricultural crops are rather widely investigated. The most suitable crops are those that offer a high, dry biomass yield at a low cost, require low nutrient and energy inputs, and do not decrease biodiversity. Among them, maize can be identified as one of the most popular plants among energy crops. What is more, maize is a highly productive crop that can be cultivated in a wide range of climatic conditions, including in northern latitude regions [6]. Also, due to the high maize biomass yield potential, methane production varies from 7500 to 10,200 m^3^ ha^−1^, i.e., an amount that no other crop has achieved so far [7].

Biomethane potential (B_POT_) assay has been recognized as a simple and reliable method to determine the biogas yield of organic substrates [8]. Currently, there are theoretical approaches offered to estimate theoretical biochemical methane potential (TBMP). For example, TBMP can be calculated after determining crop organic composition (lipids, protein, carbohydrates, and lignin content in materials) and gaining dry biomass yield data [9]. Despite the simplicity of TBMP assay, such calculations may continue for up to 2 months, and that is expensive and time-consuming. Moreover, agronomic field-based measurements, such as crop biomass collection, are often restricted by external conditions (e.g., rain events) and confined to a short period. Moreover, field measurements are usually performed only at fixed locations so sampling does not capture the spatial variability of the analyzed indicator. Therefore, innovative techniques for predicting TBMP, such as remote sensing (RS), are of great importance to reducing the cost and increasing the mapping area. Indeed, UAV platforms and related sensor technologies are part of a fast-evolving sector that is widely utilized in precision agriculture and farming. One of the most important advantages of RS is that it provides the possibility to survey large areas in a non-invasive and non-destructive way. Focusing on crop biomass and TBMP, remote sensing may provide information on plant biophysical and biochemical parameters. Therefore, alterations in biomass and TBMP caused by management and environmental factors can be detected remotely by exploiting the spectral signature of the canopies. Recently, RS technologies gained popularity among different disciplines. For example, UAVs are now widely used in precision agriculture in order to detect or monitor the biophysical and biochemical plant parameters. UAV spectral data are used to calculate vegetation indices (VIs) linked to crop parameters [10], and this correlation can be exploited to obtain prediction maps through statistical and machine learning (ML) models. Most recent studies have shown that UAV-based models are well suited for monitoring the maize biomass [11], maize nitrogen status [12], and SPAD values of maize [13], which is directly related to plant protein and oil content [14]. All the previously mentioned maize indicators are used for the estimation of TBMP; therefore, UAV techniques could be potentially used for a cheap and rapid maize yield and TBMP mapping of field experiments or even larger areas.

Recently, more research has been done on UAVs combined with ML algorithms. Due to their great computing efficiency, minimal number of required variables, and accurate findings, ML techniques have emerged as a fundamental and efficient method for modeling and extracting patterns from remote sensing data [15]. One of the advantages of ML techniques (e.g., random forest, support vector regression) is that they are usually better at handling high-dimensional data and non-linear relationships compared to traditional regression models [16]. In recent years, due to the wide use of ML techniques in agriculture, many studies dedicated to yield prediction [17], water management [18], crop recognition [19], and weed detection [20] have been carried out. When summarising these studies, it can be said that ML may not provide a universal solution for precision farming, but it enables a better determination of the analyzed indicators with minimum human intervention. In addition, the integration of UAV and ML methods increases the possibility to obtain more accurate predictions (e.g., biomass) [10]. It is clear that the assimilation of UAV and ML data may be successfully applied in agriculture; however, to the best of our knowledge, in the northern region these technologies are not fully exploited to predict theoretical biomethane potential; thus, there are only a few research-based assessments. Another issue related to UAV observation is timing, as maize‘s growth and development stages are rapid, and therefore it is essential to reduce the cost of UAV campaigns; although, they are already much cheaper and faster than the direct estimation of TBMP. Usually, during UAV-based crop monitoring studies, there are one to four UAV flights performed per growing season when high-frequency UAV datasets for maize biomass and TBMP are not sufficiently analyzed.

A study conducted by Amon et al. [21] suggested that the amount of methane that can be produced from one hectare of land greatly depends on the maize‘s dry matter accumulation. The common view is that the key factor influencing methane hectare output is the crop‘s dry matter yield. The dynamic variations in maize‘s chemical composition throughout different maturity classes and their meaning for methane output, as well as the impact of harvest time for methane output, have been well studied. For instance, Amon et al. researcher‘s study [21] determined that the highest methane yields of 7500–10,200 m^3^/ha^−1^ were achieved via maize cultivars with a high FAO number, which starts from 300 (late variety) at harvest at the R3-Milk stage. A study in Denmark [21] highlighted remarkable differences in the harvest time and the impact of cultivars on methane yield. They also established that the highest methane yield was obtained at late harvest. Another study in Germany [22] found that when growing maize for methane production, late maize cultivars had a lower concentration of fat and protein, but a higher concentration of ash and lignin, as compared with medium and early maize hybrids. It was established that despite substantially different nutrient concentrations between the maize cultivars, no clear-cut association existed between chemical composition and specific methane yield.

Therefore, the objectives of our study were: (i) to track UAV-based multispectral bands at the main phenological stages of maize, and based on these remote data, establish the correlation between VIs, TAB, and TBMP; (ii) to estimate maize total above-ground biomass and theoretical biochemical methane potential yield, combining UAV techniques with ML methods; and (iii) identify the maize phenological stage during which total-above ground biomass (TAB) and theoretical biochemical methane potential (TBMP) predictions are the most specific.

## 2. Results

### 2.1. Environmental Conditions and Maize Growth

The weather conditions during the maize growing season in 2021 were warm with optimal rainfall conditions, but there was a distinct contrast between different months, both in temperature and rainfall regime (Table 1).

During the 2021 maize sowing harvest, the mean air temperature was 17.3 °C, which was 1.5 °C above the 1991–2020 historical average. During the maize vegetative period (VE–VT), the average air temperature was 20.2 °C, which was 3.0 °C higher than the 1991–2020 historical average; however, during the reproductive period (R1–R6), the average temperature was lower compared to the vegetative period and reached 14.5 °C, which was equal to the historical average during the 1991–2020 range. The year 2021, in terms of rainfall regime, was near optimal during the entire maize growing season, and the sum of precipitation was 234.5 mm (86.2% of precipitation compared to the climate normal between 1991 and 2020). During the entire maize growing season, the number of days with heavy precipitation (above 10 mm) was 6 days. Despite the fact that the cumulative rainfall (234.5 mm) during the entire maize growing season was near optimal compared to the long-term average, contrasting differences were observed between the vegetative and reproductive development stages. For instance, during the maize vegetative development stage, the rainfall was 45.6 mm, which was only 41.1% of the precipitation norm between 1990 and 2020. According to the calculated SPI index, the maize vegetative development stage may be attributed to a moderately dry period. In contrast, a significantly higher amount of precipitation occured during the maize reproductive development stage—188.9 mm, which was 117.3% of the precipitation norm between 1990 and 2020. According to the calculated standardized precipitation index (SPI), the maize reproductive development stage may be attributed to relatively normal conditions.

Due to different maize hybrids, total above-ground biomass (TAB) varied from 9.70 t/ha^−1^ to 15.00 t/ha^−1^ (dry weight). ANOVA analysis indicated the significant (*p* < 0.05) effect of maize cultivars on TAB variation in 2021. According to the received TAB yield, maize hybrids may be grouped as follow: DKC3201 ≈ DKC2684 ≈ DKC2891 (TAB: 13.3–15.0 t ha^−1^) > DKC2978 ≈ DKC3079 ≈ DKC3204 (TAB: 12.4–12.6 t ha^−1^) > DKC3218 (TAB: 9.7 t ha^−1^).

### 2.2. Chemical Composition of Maize Samples

At maize physiological maturity, protein concentration in the TAB yield ranged from 6.6% to 8.0% [Table 2].

Performed statistical analysis indicated significant (*p* < 0.05) differences between the cultivar treatments. It has been observed that a statistically significant protein concentration was higher in the earlier maturity cultivars, i.e., DKC3218 (FAO190)—8.0% and DKC2978 (FAO190)—7.64%. Slightly lower protein concentration values were determined in the intermediate-maturity maize cultivars: DKC2684—7.56%, DKC3079—7.05%. In the remaining treatments of longer vegetation cultivars, protein concentration was below 7.0%. In all experimental treatments, lipid concentration varied within the range of 2.53–3.04%. Lipid variation was similar to protein concentration, i.e., higher lipids values were determined in earlier maturity cultivars (FAO190); however, these differences were not significant. Structural carbohydrates in the maize TAB yield varied within a rather narrow range, for instance, cellulose varied from 10.82 to 13.30%, hemicellulose from 15.78 to 16.78%, and lignin from 6.41 to 7.88%; these differences between treatments were not significant. Non-structural carbohydrates at the harvest ranged from 35.12 to 43.58%, and these differences were not significant either. However, when analyzing the total amount of carbohydrates in maize TAB, there were significant differences (*p* < 0.05) between different cultivars, and the order of treatments was as follows: DKC3079 ≈ DKC2891 ≈ DKC3204 > DKC2684 ≈ DKC3201 ≈ DKC2978 > DKC3218. It was also determined that cultivars of different maturities did not have a significant effect on the ash concentration in maize TAB.

### 2.3. Maize Yield Prediction Accuracy Using UAV-Based Multispectral Data

**Figure 1:**
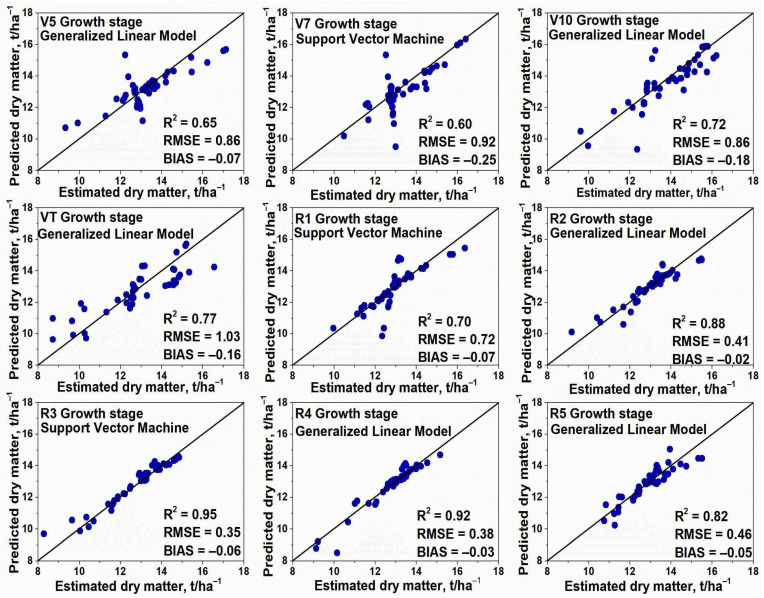
Statistical values between predicted vs. estimated total above-ground biomass (TAB) data for rainfed maize at Akademija.

The generalized linear model (GLM), a support vector machine (SVM), and random forest (RF) models were tested regarding maize TAB estimation. Figure 1 refers to the highest performing model in terms of RMSE for each growth stage.

When comparing the main maize growth and development periods, i.e., vegetative (from VE-Emergence to VT-tasseling) and reproductive (from R1-Silking to R6-physiological maturity), it can be stated that slightly better TAB prediction results were obtained during the reproductive period. At the beginning of the maize vegetative period, i.e., during the leaf formation from stage V5 to V7, statistical parameters indicated a generally good level of agreement between the observed and predicted TAB with R^2^, which varied in the range of 0.60–0.65 (*p* < 0.01); RMSE, which varied in the range of 0.86–0.92 t ha^−1^; and BIAS, which varied from −0.07 to −0.25. During later vegetative periods, i.e., at the end of the leaf development (V10) and tasseling (VT), TAB prediction results have shown a better level of agreement with R^2^ 0.72–0.77 (*p* < 0.01), RMSE 0.86–1.03 t ha^−1^, and BIAS −0.16 to −0.18. During the first maize growing season, the best TAB prediction results were demonstrated by GLM; however, the results of SVM and RF models were very similar while the differences in TAB prediction results between the different models did not exceed 9.1%, which indicates that the maize growth and development stage have a significantly greater impact on the prediction results than the selected model. During the maize reproductive stages, the model’s prediction capability consistently increased from stage R1 to R3, when the best TAB prediction results were achieved over the entire maize growing season. Then, at the R3 stage, high R^2^—0.95 (*p* < 0.01), small RMSE—0.35 t ha^−1^, and BIAS close to zero, i.e., −0.06, indicated perfect prediction results. During maize growth stage R4, TAB prediction results were slightly worse compared to R3; meanwhile, at stage R5, TAB prediction results were significantly worse than R3 or R4. During the second maize growing season, the best TAB prediction results had been determined using GLM and SVM models; however, the results of the RF model were also rather similar, and the difference between the best and the worst model in a specific maize growing period was no higher than 5.4%.

### 2.4. Maize Theoretical Biochemical Methane Potential Prediction Accuracy Using UAV-Based Multispectral Data

**Figure 2:**
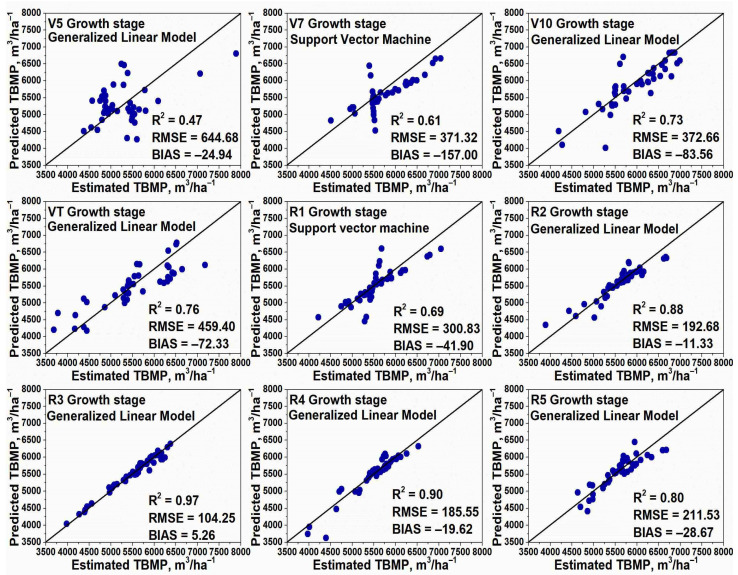
Statistical values between predicted vs. estimated theoretical biochemical methane potential (TBMP) data for rainfed maize at Akademija.

The performance results of the highest performing model in predicting maize TBMP values among GLM, SVM, and RF are shown in Figure 2 at nine growth and development stages.

The same trend discovered regarding TAB was found regarding the TBMP prediction, i.e., better TBMP prediction results were obtained during the maize reproductive period, compared to the vegetative period. Statistical analysis obviously demonstrated that it is extremely important to choose a suitable prediction period for both maize TAB and TBMP predictions. Among four maize growth and development stages in the vegetative period, the best TBMP prediction performance was determined to be during stages V10 and VT when R^2^, varied in the range of 0.73–0.76 (*p* < 0.01); RMSE, varied in the range of 372.7–459.4 m^3^ t ha^−1^; and BIAS values, varied in the range of −72.3 to −83.6, have shown a small TBMP underestimation. Meanwhile, at maize stages V5 and V7, statistical results were only reasonably good, with R^2^ 0.47–0.61 (*p* < 0.01), RMSE 371.3–644.7 m^3^ t ha^−1^, and BIAS −24.9 to −157. During the maize reproductive period, the statistical accuracy between predicted and observed TBMP values was the highest during growth stage R3; while R^2^ was high—0.97, RMSE was rather low—104.3 m^3^ t ha^−1^, and BIAS was close to zero, i.e., 5.3. Slightly worse results when predicting TBMP values were received at maize stages R2 and R4, when R2 slightly varied from 0.88 to 0.90, while RMSE varied from 185.6 to 192.7 m^3^ t ha^−1^, and BIAS varied from −11.3 to −19.6. Having compared the models used in this study, it can be stated that GLM was the most accurate model for maize TBMP prediction; however, SVM and RF models have shown slightly poorer results, meaning they can also be used for maize TBMP prediction.

### 2.5. Variable Importance (VI) Analysis

#### 2.5.1. Maize Yield

**Figure 3:**
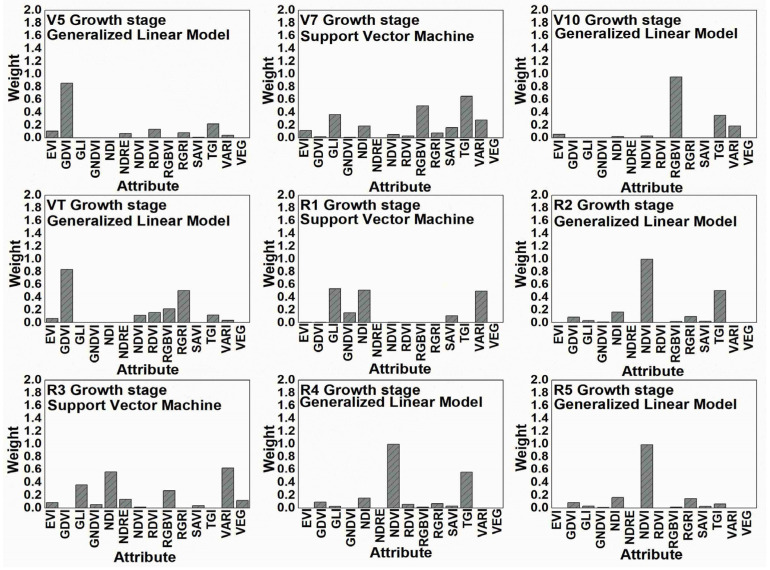
Barplots showing the weights of attribute values for maize total above-ground biomass (TAB) prediction models.

The average weights of an attribute of each calculated VI for the maize TAB, at different growth and development stages, are shown in Figure 3, according to the models shown in Figure 1.

The analysis has demonstrated the importance of maize growth and development stages as covariates in the use of models for TAB prediction. No clear trends have been found when analyzing the distribution of different VI weights among different maize growth and development stages. For instance, GDVI VI showed very high weight values at stages V5 and VT, but at stages V10, R1, and R3, the weight values were 0. Similar results were obtained using other VIs, for example, NDVI weight values were very high during the reproductive period, i.e., stages R2, R4, and R5; however, during the vegetative period, the results were the opposite. In general, all of the weight values exhibited high variability across the nine growth stages. For maize TAB prediction, VIs may be grouped according to their importance as follow: NDVI ≈ TGI ≈ GDVI ≈ RGBVI > NDI ≈ VARI ≈ GLI ≈ RGRI > EVI ≈ RDVI ≈ SAVI ≈ GNDVI ≈ NDRE ≈ VEG. It should be noted that the VI of the last group (VEG) had almost no effect on the maize TAB prediction. To summarise the results, the estimated and used UAV-based VIs as input data for ML models were different for each maize growth stage, indicating that the suitable UAV covariates for the TAB prediction were greatly affected by the maize growth stage.

#### 2.5.2. Maize Theoretical Biochemical Methane Potential

**Figure 4:**
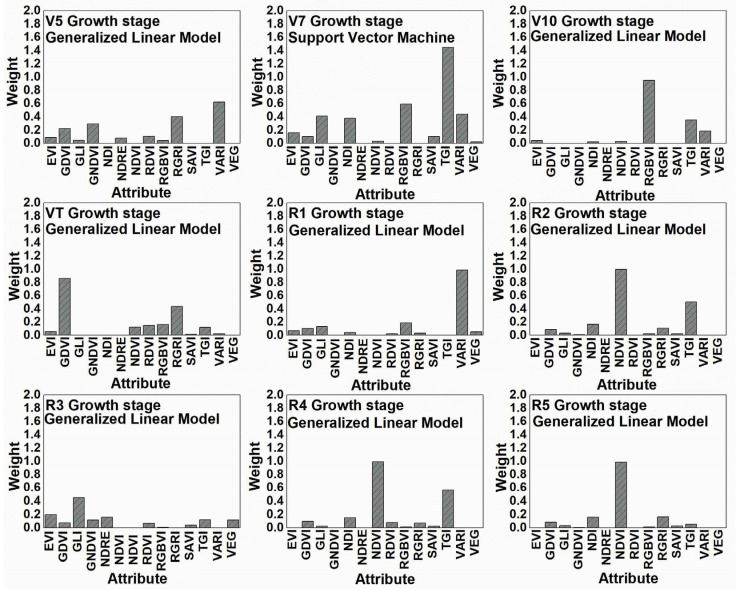
Barplots showing the weights of attribute values for maize theoretical biochemical methane potential (TBMP) prediction models.

The weights of the attributes which reflect the relative importance of the 14 VI used in this study for maize TBMP prediction are shown in Figure 4, according to the models shown in Figure 2.

For maize TBMP estimation models, the greatest covariates were NDVI (especially R2 and R4-R6 stages) and TGI (especially V7, R2, and R4 stages); however, it should be noted that in some maize growth stages, the aforementioned VIs did not have a significant impact on maize TBMP prediction. On the contrary, the lowest weight values were obtained using NDRE and VEG indices. To summarise the results, the VIs can be arranged in the following order of importance: NDVI ≈ TGI ≈ VARI > RGBVI ≈ GDVI ≈ RGRI ≈ GLI ≈ NDI > EVI ≈ GNDVI ≈ RDVI ≈ SAVI ≈ VEG ≈ NDRE. When predicting maize TBMP as well as TAB values, a similar trend remained, i.e., the last group VI (VEG) had almost no effect. To summarise the results, it can be stated that the tendencies remained the same as they were during TAB prediction, i.e., UAV covariates for TBMP prediction were considerably affected by maize growth stages.

### 2.6. Maize Yield and Theoretical Biochemical Methane Potential Mapping

**Figure 5:**
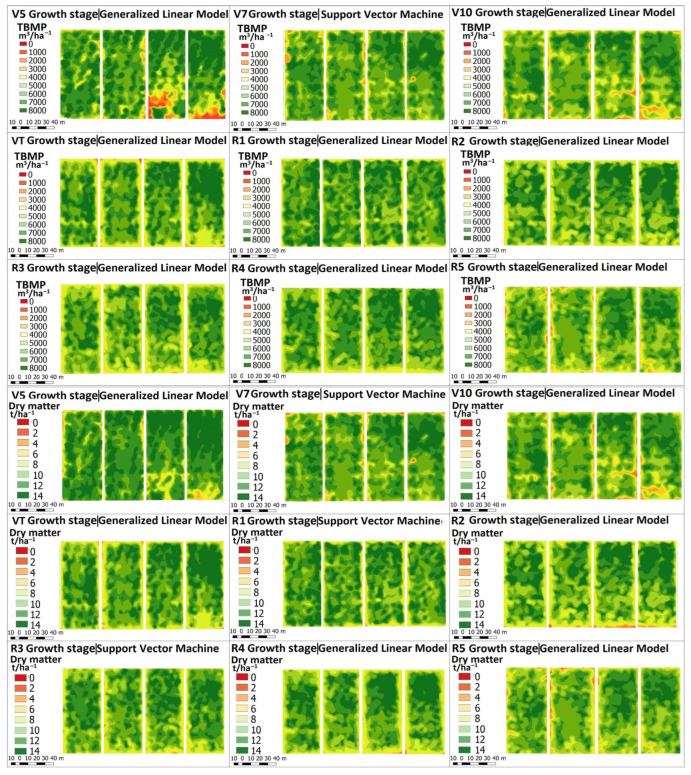
The heat map of estimated maize total above-ground biomass as dry matter and theoretical biochemical methane potential (TBMP).

As shown in Section 2.3 and Section 2.4, the models with the highest prediction accuracy were selected, and based on these results, the TAB and TBMP maps of the predicted maize conditions at nine growth and development stages were developed (Figure 5). As shown in Figure 5, clear differences in the spatial distribution of TAB and TBMP values are seen among maize hybrids across growth stages.

## 3. Discussion

Our study provides experimental evidence regarding different maize hybrids and their total above-ground biomass levels, which varied significantly from 9.70 t/ha^−1^ to 15.00 t/ha^−1^ (in dry weight), in a region in the Baltic area. Due to the simplicity, easy interpretability, and high acceptance of the RS approach, we obtain high-resolution UAV-based multispectral band data, which have been used for maize TAB and TBMP prediction. Although the VI-based TAB estimation method is considered to be very efficient, the remotely obtained maize TAB and TBMP data cannot effectively describe the mechanistic response of changes in maize growth and development in different environments or under different agronomic management practices. For this reason, 14 VIs derived from UAV-based multispectral images were employed. In addition, some studies demonstrated that the growth stage plays a key role in the sensitivity and performance of VIs when predicting crop biophysical parameters [23]; there is also a lack of comprehensive high-frequency UAV observation for maize. Therefore, in our study, the UAV campaign was carried out nine times during all main growth stages of maize. A detailed study of maize yield prediction at different physiological stages was carried out by Barzin et al. [24]; they discovered that VI-based prediction models at maize stages V10 and VT had the greatest accuracy with R^2^ values of 0.90 and 0.93, respectively, while in our study, prediction accuracy during the same period was slightly lower, with the R^2^ values being 0.73 at V10 and 0.76 at VT. However, the results of both studies confirmed that during the initial maize growth stages (V3–V7), TAB prediction accuracy was lower than it was during later growth periods (V10–VT). In contrast to that, a study by Zhang et al. [11] has shown that, through using the hyperspectral imageries of maize canopy and measuring maize height data, the stepwise regression model managed to explain 85–87% of TAB variation at the V6, R1, and R3 growth stages; this showed that the maize growth stages did not have such a significant impact on prediction accuracy as what is implied in the above-mentioned studies. Another study by Ji et al. [25] presented detailed research on maize yield prediction that used phenological information from RS; the researchers divided the maize growing season into four growth phases, i.e., 1—from V1 to V6, 2—from V6 to VT, 3—from VT to R4, and 4—from R4 to R6. It was found that the phenological phases have a significant effect on the prediction of maize yield, and the period from maize flowering (VT) to dough (R4) is the most optimal period for yield prediction. These results are in line with the results we obtained, i.e., the best TAB prediction accuracy was found during the R2–R4 growth stage period when R^2^ varied from 0.88 to 0.95. The silking-dough (R1–R4) stages represent the nutrient (nitrogen, phosphorus, potassium) concentration growth peak of the maize parts of the leaf, stem, and storage organs (shank, cob), while nutrient concentration in the grain only begins to increase during this period [26]. During the R1–R4 period, maize has the highest LAI [27] while the leaves are still green, which is probably the reason why the best prediction results were obtained during the maize silking-dough period. However, these comparisons between the different maize yield prediction studies should be treated carefully, as, in many cases, different genotypes have been grown and different management practices have been used in different soil types and climatic conditions, which can possibly have a great impact on the accuracy of the prediction. According to one study [26], a rapid increase in maize kernel weight starts during period R2–R4, and the results of our study suggest that this period is the most suitable for maize yield prediction within the Nordic-Baltic region. Although stage R3 is a perfect period for yield prediction, for farmers, it may be a bit too late as it is close to the end of the maize life cycle, and it is therefore practically impossible to make any agronomic management changes to increase yield. Thus, we determined that the earliest suitable observing period for maize yield prediction in the region is V7–V10 because, at that time, the accuracy of the prediction is sufficiently high and the N demand is still low; therefore, the last N fertilization is still possible. At very early maize growth stages (V5), TAB prediction accuracy was the lowest (R^2^ = 0.65) among all of the growth stages analyzed. The results were largely to be expected, as during the initial maize growth stages, the demand for water and nutrients was rather low; thus, the difference in maize hybrids was not so notable. As a result, ML models during those growth stages could not represent well enough the TAB differences caused by the various maize hybrids. On the contrary, maize nutrient demand starts to increase rapidly from around stage V7, and at the end of the vegetative period (V10–V14), the differences are clearly notable [28]. During a similar time, maize also reached its water demand peak, which happens during flowering and at the early grain-filling period [26]. In addition to that, it has been determined that relevant condition when developing data-driven prediction models for yield is when RS data is obtained during the crop growth stage, which is crucial in kernel growth [29]. It is important to note that even though different maize cultivars (FAO190-230) have been used in our study, they all had a “stay-green” effect, which resulted in the leaf senescence being almost the same in all of the cultivars. It is likely that mainly because of this, different cultivars did not have a significant effect on the prediction results.

Maize is a well-known crop used in methane production and can produce between 7500 and 10,200 m^3^ ha^−1^ under suitable climate conditions [7]. However, the climatic conditions for maize production in the Nordic-Baltic region are still marginal as the crop is exposed to cold weather and water stresses, which mainly result in a large year-to-year variation [30]. As has been already determined, methane production output is primarily influenced by the dry matter yield of the crop, while the chemical composition of the crop has a significantly lesser influence on the amount of methane production [22]. As a result, during maize harvesting, the estimated TBMP levels in our experiment ranged from 4092 to 6626 m^3^ ha^−1^ and the correlation between TAB and TBMP was R^2^—0.98, indicating that TAB is the main factor that mainly determines TBMP levels. This finding, which revealed a strong relationship between TAB and TBMP, was also responsible for the fact that the accuracy of TBMP prediction at different maize growth stages was very similar to that of TAB values. The most accurate TBMP values could be predicted at the R3 and R4 growth stages where R^2^ was 0.90 and 0.97, respectively. These results nicely coincided with the findings of Amon et al. [7] who suggested that maize should be harvested at growth stages R3–R4 to achieve the highest methane yield. However, we could not find similar studies where UAV-based multispectral data were used for TBMP prediction.

To acquire the most suitable model for predicting maize TAB and TBMP, we used three ML methods, i.e., GLM, RF, and SVM models. When comparing the performances of these models during each maize growth stage, the accuracy of all of the used models’ predictions was very similar; although, slightly better results regarding TAB and TBMP prediction were obtained with the GLM and SVM models. However, the differences in the different models’ predictions were not so evident, and thus, all three models used in our study may be successfully employed when predicting maize TAB and TBMP values under Nordic-Baltic climate conditions.

When predicting the TAB and TBMP values, it has been observed that when using only spectral bands (e.g., red, green, blue), the accuracy of the predictions was not sufficient. Thus, in the further steps of this study, only calculated VIs were used and their possible link with dependent TAB and TBMP variables was sought. However, even when using different VIs for maize prediction, no clear insights have been found. For example, during the maize vegetative period for TAB prediction, GDVI performed relatively better than the other VIs at the V5 and VT stages, but later, at the V10, R1, and R3 stages, there was almost no weight effect on the prediction. Similar trends remained when using the other VIs, for example, NDVI was very important at R2, R4, and R5 stages, but during the vegetative period, the results were the opposite. It is only important to note that at that time, i.e., from the R2 to R5 stages, the NDVI values were the highest during the whole maize growing season and varied within the range of 0.68 to 0.73, while the canopy was closed and the NDVI saturated. Another interesting observation is that at the R3 stage, i.e., the time when the accuracy of the TAB predictions was the best, the GLI, NDI, and VARI indices had the greatest impact on these predictions and all of them have a green band in their formulas, as well as not having an NIR band. It has also been noted that most of the VIs used were saturated at the R3 stage of maize growth. When analyzing the TBMP predictions, the trends were very similar to the TAB, i.e., different VIs at different maize growth stages had different effects on the accuracy of the predictions. Certainly, it was greatly affected by the fact that we used different ML models for the prediction; therefore, VI weights at different maize growth stages should be compared carefully.

RS technologies, more precisely, UAVs at low altitudes, have important application options for acquiring crop spectral information at the plot scale. One of the greatest advantages of using UAVs compared to satellites is that it is possible to compensate for the lack of spatial and temporal resolution of satellite data in precision agriculture. In this study, we tried an exploration in predicting maize TAB and TBMP, and this is an initial attempt, at least in regards to the Nordic-Baltic area. Our prediction results were rather good, but they could be further improved and more studies that cover different soil types and environmental conditions are required. Our research results are based on a dataset of one-year field experiments under typical weather conditions that prevailed in the area. Thus, this deficiency should be corrected by future multi-year field experiments, testing how TAB and TBMP change under contrasting weather conditions. What is more, the use of more data from field seasonal measurements (e.g., chlorophyll content, LAI, etc.), as well as climatic and management data, could provide a more detailed description of crop growth and allow for more favorable predictions of TAB and TBMP.

## 4. Materials and Methods

### 4.1. Study Site and Maize Experiment

**Figure 6:**
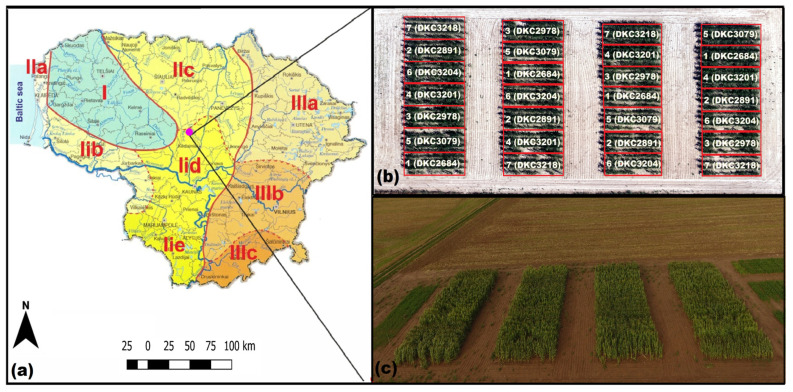
(**a**)—Lithuanian agroclimatic zones, the numbers (I, II, III) and the letters (a, b, c, d, e) denote districts and subdistricts of agroclimatic zones, respectively, the purple dot marks weather station location; (**b**)—The arrangement of field experiment treatments in randomized block design with four replicates; (**c**)—aerial image of maize field experiments at milk growth and development stage.

According to the Köppen climate classification [31], the climate of Lithuania is humid continental (Dfb), with warm summers and rather severe winters. The territory of Lithuania is not homogeneous regarding air and soil temperature, precipitation distribution, and soils. The average annual air temperature ranges between 5.8 and 7.6 °C, and annual precipitation between 550 and 910 mm, of which 60 to 66% falls in the April–October period. The main soils are Luvisols, Cambisols, Gleysols, Arenosols, Retisols, and Histosols and they cover 28.5, 15.9, 14.6, 13.2, 9.4, and 8.5% of the area, respectively. Field experiments with maize were carried out in 2021 at the Lithuanian Research Centre for Agriculture and Forestry, located in Akademija (55°23′09″ N 23°52′41″ E). The field experiment locations fell into the agro-climatic zone IID (see Figure 6) of central Lithuania [32], which is warm but also the driest compared to other zones.

The maize (*Zea mays* L.) field experiment under rainfed conditions was conducted during the 2021 season at the Lithuanian Research Centre for Agriculture and Forestry (55°23′50′′ N and 23°51′40′′ E) in the Central part of Lithuania. The main soil was Endocalcari–Epihypogleyic Cambisol (WRB, 2014) with loam texture class (49% Sand, 35.4% Silt, 15.6% Clay); it had a neutral pH of 6.8 and soil organic matter of 3.2%. In this study, seven maize hybrids with different maturity classes and different suitability levels (grain, silage, biogas) were investigated (Table 3). These maize varieties were selected based on the high amount of dry matter and their suitability to be grown in this region.

The maize was grown after conventional tillage and was sown on 30 May 2021, when soil temperature had reached 10–12 °C, with a density of 90,000 plants ha^−1^ (0.75 m row and 0.15 m plant spacing). Weeds were controlled by the herbicide Arrat (tritosulphurone 250 g/kg^−1^ + dicamba 500 g/kg^−1^) and commercial formulation wettable granule (WG) at a rate of 0.2 kg/ha^−1^ was used at the maize V3 growth stage. During the maize growing season, no diseases were observed; thus, other pesticides were not used. All experimental treatments were fertilized according to local practices and followed the same rates as those of mineral fertilizers. Before maize sowing; nitrogen (N), at a rate of 120 kg N ha^−1^; superphosphate (P), at a rate of 90 kg P ha^−1^; and potassium chloride (K), at a rate of 170 kg K ha^−1^ were applied manually and incorporated into the soil. Additionally, at the growth stage of V5, maize was fertilized with N 80 kg N ha^−1^ to avoid N deficiencies. The mineral fertilizers were in the form of ammonium nitrate (AN) (34.4–0–0), while complex NPK fertilizers were in the form (6–18–34). Maize harvest was performed manually on 6 October 2021. The field experiments included seven treatments that were performed in randomized block design with four replicates. The area of each experimental plot was 27 m^2^.

### 4.2. Plant and Soil Measurements

During the maize vegetation period, plant development stages were recorded frequently. The maize vegetative and reproductive development stages were identified on the basis of the entire treatment when 50% or more of the plants were at a particular development stage. The leaf-collar method [26] was used for the development of vegetation stages, whereas reproductive stages were based on established visual indicators of kernel development. At physiological maturity, when more than 50% of the plants showed a visible black layer at the base of the kernel, four rows of each plot from an area of 8 × 3.0 = 24 m^2^ were cut to identify the final total aboveground biomass and grain yield. The individual maize components were weighed (fresh mass) and dried at 65 ± 5 °C to constant weight (dry weight).

A modern, environmentally friendly analysis method, near-infrared (NIR) spectroscopy, which does not require chemical reagents, was used to examine the quality of the maize samples. Maize TAB samples were scanned with a NIRS-6500 device, with a spinning module using wavelengths between 400 and 2500 nm in reflectance, and the obtained spectra were processed with equations installed into the device for maize analysis (VDLUFA Laboratory, Speyer, Germany). For analysis, the samples were dried and ground with an ultra-centrifugal mill ZM 200 (Retsch, Haan, Germany) to pass a 1 mm screen. The precision of the used equations was sufficient and ranged within the following limits: standard error calibration (SEC)—0.16–0.64, coefficient of determination (RSQ)—0.77–0.94, and standard error cross-validation (SECV)—0.18–0.68. When using this method, maize quality parameters, including protein, lipid, and ash contents, have been determined. The number of water-soluble carbohydrates was determined by Anthron’s method.

Soil samples were collected before the implementation of the experiment and they were later air-dried and passed through 2 mm and 0.25 mm size sieves in the laboratory. SOC content was determined by a photometric procedure at a wavelength of 590 nm using a UV–VIS spectrophotometer Cary (Varian) and using glucose as a standard [33]. The soil texture fraction was determined using the pipette method. All chemical analyses were conducted in the Chemical Research Laboratory, while texture analyses were carried out in the Department of Soil and Crop Management of the Lithuanian Research Centre for Agriculture and Forestry.

### 4.3. Theoretical Methane Yield

In this study, we calculated theoretical biochemical methane potential (TBMP) based on the maize sample’s organic composition (expressed as *TBMP_org_*). *TBMP_org_* was calculated by Equation (1) [9],

(1) $$TBMP_{org}\left(\frac{mLCH_{4}}{g\;VS}\right)=\frac{373VFA+496Protein+1014Lipids+415Carbohydrates+727Lignin}{100}$$

where volatile fatty acid (*VFA*), lipids, protein carbohydrates, and lignin were expressed in terms of %. In the next step, TBMP values were recalculated into m^3^/ha^−1^ units.

### 4.4. Remote Data Acquisition

The UAV system for the image collection of the field experiment was a consumer-grade quadrotor Phantom 4 Professional (SZ DJI Technology Co., Shenzhen, China) with an installed real-time kinematic (RTK) module that enhances the precision of position data, derived from satellite-based positioning. Additionally, this used UAV had a sunlight sensor that automatically adjusts radiation reflectance and obtains reflectance data directly. This UAV had a combined multispectral imaging system, including a visible light (RGB) sensor responsible for visible light imaging. There are five additional multispectral bands with 5.74 focal lenses, including blue light (B), green light (G), red light ^®^, red edge, and near-infrared (NIR), with center wavelengths of 450, 560, 650, 730, and 840 nm, respectively. The UAV campaign was carried out 9 times during the main maize growth stages (Table 4) at a height of 25 m above ground level.

The UAV flights were conducted in clear sky and low wind speed conditions between 11:00 am and 13:00 pm local time. The UAV flights were controlled using the flight planner app (Pix4D SA, Lausanne, Switzerland), adapted for android OS and using the Huawei P20 smartphone. The average speed of each UAV flight was ≈2.2 m/s, with a camera looking downwards; the flight duration for the full experimental plot cover lasted approximately 20 min; and about 2100 images, with 85% vertical and 85% horizontal overlapping to obtain images with a 1.1 pixel size, were taken during each flight.

### 4.5. UAV Image Processing and Calculation of VIs

After each flight UAV, multispectral image data was preprocessed with Pix4D software, which uses the structure-from-motion technique. This technique was used to relate features between overlapping images and calculate the 3D position of the matched points, which are densified and textured with the corresponding images. The ortho-mosaic image was generated by projecting each texture point onto the 2D plane and was exported in a TIFF image format for further analysis. After the image stitching process, the generated ortho-mosaic ground sampling distance was 1.09 cm/pixel. Then, the densified point clouds were generated with 5,049,015 points. The RMSE values in the X, Y, and Z coordinates were 0.020 m., 0.017 m., and 0.052 m., respectively. In order to calculate the vegetation indices (VI) consisting of different combinations of wavelength-specific spectral reflectance, the prepared ortho-mosaic imagery was imported to freely available QGIS software (version 3.2.2.). In the next step, experimental plots (replicates) were marked out with polygons on the ortho-mosaic image, and the vegetation indices were calculated using the raster calculator tool. In this study, we computed 14 widely used VIs for predicting maize TAB and TBMP. The majority of the selected VIs had been used in previous studies for monitoring the growth and predicting the yields of agricultural crops. Based on this, we selected the most commonly used VIs and they were tested in our study (Table 5).

### 4.6. Machine-Learning Methods for Predicting Biomass and Theoretical Methane Yield

In this study, we used three widely used machine-learning (ML) methods, i.e., generalized linear model (GLM), random forest (RF), and Support Vector Machine (SVM). In order to predict the maize total above-ground biomass (TAB) and theoretical biochemical methane potential (TBMP), a dataset regarding each of the vegetative (V5, V7, V10, VT) and reproductive (R1, R2, R3, R4, R5) period stages, where TAB and TBMP were assigned as dependent variables and VIs as independent variables (Table 5), was divided into two parts: 60% of the dataset was used for training while the remaining 40% of the dataset was used for testing. For dataset splitting, we used the split validation method, which randomly splits up the data into a training set and a testing set. The entire prediction analysis was done using a computer that has an Intel i9 processor and 64 GB memory with a 64-bit operating system. ML prediction algorithms were created and developed with open-source data software RapidMiner Studio 9.9 (RapidMiner Inc., Boston, MA, USA).

Generalized linear models (GLM) are an extension of traditional linear models [47]. This algorithm fits generalized linear models to the data by maximizing the likelihood of the training parameters. The model fitting computation is parallel, fast, and scales well for models with a limited number of predictors.

A random forest (RF) is an ensemble of a certain number of random trees and is more robust with respect to noise [48]. These trees are created/trained on bootstrapped sub-sets provided by the input data. Each node of a tree represents a splitting rule for one specific attribute. Only a sub-set of attributes, specified with the subset ratio criterion, is considered for the splitting rule selection. This rule separates values in an optimal way for the selected parameter criterion. For classification, the rule is separating values belonging to different classes, while for regression, it separates them in order to reduce the error made by the estimation. The building of new nodes is repeated until the stopping criteria are met.

The Support Vector Machine (SVM) is a form of supervised nonparametric modeling, which is defined by using kernels and operating on the margins [49]. The algorithm takes a set of input data and predicts, for each given input, which of the two possible classes comprises the input, making the SVM a non-probabilistic binary linear classifier. Given a set of training examples, each marked as belonging to one of two categories, an SVM training algorithm builds a model that assigns new examples to one category or the other. An SVM model is a representation of the examples as points in space, mapped so that the examples of the separate categories are divided by a clear gap that is as wide as possible. New examples are then mapped into that same space and predicted to belong to a category based on which side of the gap they fall on.

The agreement between the observed maize TAB, estimated theoretical methane yield, and predicted parameters was tested by the coefficient of determination (R^2^), BIAS, and RMSE, computed as follows [50]:

(2) $$R^{2}=\left(\frac{\sum_{i=1}^{n}\left(y_{i}-\bar{y}\right)\left(x_{i}-\bar{x}\right)}{\sqrt{\sum_{i=1}^{n}{\left(y_{i}-\bar{y}\right)}^{2}\;\sum_{i=1}^{n}{\left(x_{i}-\bar{x}\right)}^{2}\;}}\right)$$

(3) $$\mathrm{BIAS}=1/n\sum_{i=0}^{n}\left(y_{i}-x_{i}\right)$$

(4) $$\mathrm{RMSE}=\sqrt{\frac{1}{n}\overset{n}{\underset{i=1}{\sum }}{\left(y_{i}-x_{i}\right)}^{2}}$$

where *n* is the number of observed values, *y_i_* and *x_i_* are the simulated and observed values, and $\bar{y}$ and $\bar{x}$ are the average observed and simulated values for the *i*th data pair. R^2^ describes the proportion of the variance in the observed data as explained by the prediction model, and it ranges from 0 to 1, with higher values indicating less error variance. The BIAS measures the average difference between the observed/estimated and predicted values. A positive BIAS value indicates an under-prediction and a negative BIAS indicates an over-prediction. The RMSE is the square root of the mean square error. The smaller the BIAS and RMSE values are, the better the performance of the prediction.

Significant differences between the treatments were determined using Tukey’s test at a 0.05 probability level. Statistical analyses were performed using proc GLM, SAS v9.4 (SAS Institute Inc., Cary, NC, USA).

### 4.7. Weather Data

The daily meteorological data, including mean temperature °C, minimum and maximum temperatures °C, mean humidity (%), and sunshine hours per day, were obtained from a meteorological station of the Lithuanian Hydrometeorological Service (Ministry of Environment), located near the maize experimental field.

## 5. Conclusions

In this study, we investigated the feasibility of using UAV-multispectral images in combination with field measurements and ML algorithms to estimate maize TAB and TBMP values at main phenological stages. Our study suggests that the accuracy of both TAB and TBMP prediction was greatly affected by different maize growth stages; however, different maize hybrids did not have a significant effect on prediction accuracy. The best ML prediction results were obtained during the R2–R4 maize growth period; the prediction models managed to explain 88–95% of TAB and 88–97% TBMP variation. We also found that, for the practical usage of farmers, the earliest suitable timing for TAB and TBMP prediction in the Nordic-Baltic area is within stages V7–V10. In this study, three ML algorithms were used when predicting TAB and TBMP values. The best maize TAB and TBMP prediction accuracy results were achieved using the GLM model.

## Figures and Tables

**Table 1:** Ten-day period weather conditions per maize vegetative (V) and reproductive (R) development stages in 2021. Maize growth and development stages: V5—Fifth leaf, V7—Seventh leaf, V10—Tenth leaf, VT—tasseling, R1—Silking, R2—Blister, R3—Milk, R4—Dough, R5—Dent, R6—Physiological maturity.

Month	May			June			July			August			September			October		
Day of month	^10^	^20^	^30^	^10^	^20^	^30^	^10^	^20^	^30^	^10^	^20^	^30^	^10^	^20^	^30^	^10^	^20^	^30^
Maize development						V5	V7	V10	VT	R1/R2	R3		R4		R5	R6		
Mean air temperature, °C	15.9	13.3	13.1	18.1	18.6	22.2	23.2	24.3	21.1	17.3	17.1	14.6	13.4	11.9	9.6	9.3	6.8	8.0
Minimum air temperature, °C	0.4	7.1	5.9	8.4	7.8	13.1	15.0	12.7	12.2	9.5	10.7	6.9	8.2	3.7	1.8	−2.7	−0.5	−0.3
Maximum air temperature, °C	23.8	25.6	20.9	26.2	31.3	32.1	33.0	35.0	32.2	26.3	27.3	22.2	19.6	26.7	18.2	17.1	13.8	15.3
Mean soil temperature at 15 cm, °C	8.2	13.9	12.9	18.8	20.4	23.6	23.7	25.1	23.5	19.5	17.7	15.9	13.9	13.5	10.5	9.9	7.6	7.7
Precipitation, mm	33.1	33.2	34.6	2.4	5.5	22.2	9.1	5.9	6.1	31.8	63.1	55.9	1.3	15.6	11.3	4.3	9.8	20.9
Standardized precipitation index	1.78 (Very wet)			−1.01 (Moderately dry)			−1.28 (Moderately dry)			1.71 (Very wet)			−0.61 (Near normal)			−0.21 (Near normal)		
Mean relative humidity, %	69	71	72	61	67	73	68	65	62	79	79	83	73	81	83	72	85	84
Sunshine, hour/day	7.6	7.4	7.9	11.8	12.5	10.7	10.9	10.6	11.0	5.7	5.1	4.7	8.9	3.5	3.7	7.3	3.4	4.7

**Table 2:** Chemical composition of different maize samples used in this study.

Hybrid	Proteins, %	Lipids, %	Structural Carbohydrates, %			Non-Structural Carbohydrates, %	Total Carbohydrates, %	Ash, %
			Cellulose	Hemicellulose	Lignin			
DKC2684	7.56 bac	2.84 a	10.82 a	15.78 a	6.41 a	43.58 a	76.58 ba	4.38 b
DKC2891	6.89 bc	2.53 a	11.73 a	16.30 a	6.95 a	42.83 a	77.80 a	4.46 b
DKC3079	7.05 bac	2.65 a	11.49 a	16.78 a	6.81 a	42.78 a	77.85 a	4.73 b
DKC3201	6.59 c	2.53 a	13.30 a	15.78 a	7.88 a	38.56 a	75.51 ba	4.78 b
DKC2978	7.64 ba	2.90 a	10.86 a	15.90 a	6.44 a	42.17 a	75.37 ba	4.74 b
DKC3204	6.76 bc	2.68 a	11.05 a	16.10 a	6.55 a	43.53 a	77.23 a	4.55 b
DKC3218	8.00 a	3.04 a	13.20 a	16.73 a	7.82 a	35.12 a	72.87	5.71 a

Note. Different combinations of letters indicate significantly different means (*p* < 0.05, Tukey’s test).

**Table 3:** Maize hybrids and their main characteristics used in the study.

Hybrid	Maturity	Suitable for	Type of Grain	Breeding Company
DKC2684	FAO 210	Grain, Silage, Biogas	Flint/Dent	Dekalb
DKC2891	FAO 220	Silage	Flint/Dent	Dekalb
DKC3079	FAO 230	Grain, Biogas	Dent	Dekalb
DKC3201	FAO 220	Silage, Biogas	Flint/Dent	Dekalb
DKC2978	FAO190	Silage, Biogas	Flint/Dent	Dekalb
DKC 3204	FAO 230	Grain, Silage, Biogas	Flint/Dent	Dekalb
DKC3218	FAO 190	Silage, Grain	Flint/Dent	HR Smolice

**Table 4:** Summary of the UAV flights in the corresponding maize growth stages in 2021.

Code	Common Name	Sowing/Harvest Date	Date of UAV Flights	Days after Planting
	Planting	30 May 2021		0
V5	Fifth leaf		25 June 2021	26
V7	Seventh leaf		3 July 2021	34
V10	Tenth leaf		17 July 2021	48
VT	Tasseling		25 July 2021	56
R1	Silking		1 August 2021	63
R2	Blister		8 August 2021	70
R3	Milk		17 August 2021	79
R4	Dough		5 September 2021	98
R5	Dent		25 September 2021	118
R6	Physiological maturity	6 October 2021		129

**Table 5:** Vegetation indices (VI) calculated in the study in order to test the usefulness of multispectral aerial images to predict maize total above-ground biomass and theoretical biochemical methane potential.

	Vegetation Index (VI)	Name	Formula	Reference
1.	EVI	Enhanced Vegetation Index	2.5 × (NIR − Red)/(NIR + 6Red − 7.5Blue + 1)	[34]
2.	GDVI	Green Difference Vegetation Index	NIR − Green	[35]
3.	GNDVI	Green Normalized Difference Vegetation Index	(NIR − Green)/(NIR + Green)	[36]
4.	NDRE	Normalized Difference Red-edge	(NIR − Red-edge)/(NIR + Red-edge)	[37]
5.	NDVI	Normalized Difference Vegetation Index	(NIR − Red)/(NIR + Red)	[38]
6.	RDVI	Renormalized Difference Vegetation Index	$(\mathrm{NIR}-\mathrm{Red})/\sqrt{\mathrm{NIR}+\mathrm{Red}}$	[39]
7.	SAVI	Soil-Adjusted Vegetation Index	1.5 × (NIR − Red)/(NIR + Red + 0.5)	[40]
8.	GLI	Green leaf index	GLI = (2 × Green − Red − Blue)/(2 × Green + Red + Blue)	[41]
9.	NDI	Normalized difference index	NDI = (Green − Red)/(Green + Red)	[42]
10.	RGBVI	Red Green Blue Vegetation Index	RGBVI = (Green2) − (Blue × Red)/(Green2) + (Blue × Red)	[43]
11.	RGRI	Red-Green Ratio Index	RGRI = Red/Green	[44]
12.	TGI	Triangular greenness index	TGI = Green − 0.39 × Red − 0.61 × Blue	[45]
13.	VARI	Visual atmospheric resistance index	VARI = (Green − Red)/(Green + Red − Blue)	[46]
14.	VEG	Vegetative	VEG = Green/Redα × Blue1−α, α = 0.667	[40]

## References

[1] Li Y., Zhang R., Liu G., Chen C., He Y., Liu X. (2013). Comparison of methane production potential, biodegradability, and kinetics of different organic substrates. Bioresour. Technol..

[2] Gunaseelan V.N. (1997). Anaerobic digestation of biomass for methane production: A Review. Biomass Bionergy.

[3] Möller K., Stinner W. (2010). Effects of organic wastes digestion for biogas production on mineral nutrient availability of biogas effluents. Nutr. Cycl. Agroecosyst..

[4] Uusitalo V., Havukainen J., Manninen K., Höhn J., Lehtonen E., Rasi S., Soukka R., Horttanainen (2014). Carbon footprint of selected biomass to biogas production chains and GHG reduction potential in transportation use. Renew. Energy.

[5] Raposo F., De la Rubia M.A., Fernández-Cegrí V., Borja R. (2012). Anaerobic digestion of solid organic substrates in batch mode: An overview relating to methane yields and experimental procedures. Renew. Sustain. Energy Rev..

[6] Žydelis R., Weihermüller L., Herbst M. (2021). Future climate change will accelerate maize phenological development and increase yield in the Nemoral climate. Sci. Total Environ..

[7] Amon T., Amon B., Kryvoruchko V., Machmüller A., Hopfner-Sixt K., Bodiroza V., Hrbek R., Friedel J., Pötsch E., Wagentristl H. (2007). Methane production through anaerobic digestion of variuous energy crops grown in sustainable crop rotations. Bioresour. Technol..

[8] Labatut R., Angenent L.T., Scott N.R. (2011). Biochemical methane potential and biodegradability of complex organic substrates. Bioresour. Technol..

[9] Triolo J.M., Sommer S.G., Møller H.B., Weisbjerg M.R., Jiang X.Y. (2011). A new algorithm to characterize biodegradability of biomass during anaerobic digestion: Influence of lignin concentration on methane production potential. Bioresour. Technol..

[10] Li D., Miao Y., Gupta S.K., Rosen C.J., Yuan F., Wang C., Wang L., Huang Y. (2021). Improving Potato Yield Prediction by Combining Cultivar Information and UAV Remote Sensing Data Using Machine Learning. Remote Sens..

[11] Zhang Y., Xia C., Zhang X., Cheng X., Feng G., Wang Y., Wang Y., Gao Q. (2021). Estimating the maize biomass by crop height and narrowband vegetation indices derived from UAV-based hyperspectral images. Ecol. Indic..

[12] Guo Y., Wang H., Wu Z., Wang S., Sun H., Senthilnah J., Wang J., Bryant C.R., Fu Y. (2020). Modified Red Blue Vegetation Index for Chlorohyll Estimation and Yield Prediction of Maize from Visible Images Captured by UAV. Sensors.

[13] Guo Y., Chen S., Li X., Cunha M., Jayavelu S., Cammarano D., Yongshuo F. (2022). Machine Learning-Based Approaches for Predicting SPAD Values of Maize Using Multi-Spectral Images. Remote Sens..

[14] Iqbal S., Thierfelder C., Khan H.Z., Javeed H.M.R., Arif M., Shehzad M. (2017). Maximizing maize quality, producttivity and profitability through a combined use of compost and nitrogen fertilizer in a semi-arid environment in Pakistan. Nutr. Cycl. Agroecosyst..

[15] Ali I., Greifeneder F., Stamenkovic J., Neumann M., Notarnicola C. (2015). Review of Machine Learning Approaches for Biomass and Soil Moisture Retrievals from Remote Sensing Data. Remote Sens..

[16] Hansen P.M., Schjoerring J.K. (2003). Reflectance measurements of canopy biomass and nitrogen status in wheat crops using normalized difference vegetation indices and partial least squares regression. Remote Sens. Environ..

[17] Chlingaryan A., Sukkarieh S., Whelan B. (2018). Machine learning approaches for crop yield prediction and nitrogen status estimation in precision agriculture: A review. Comput. Electron. Agric..

[18] Virnodkar S.S., Pachghare V.K., Patil V.C., Jha S.K. (2020). Remote sensing and machine learning for crop water stress determination in various crops: A critical review. Precis. Agric..

[19] Pushpanathan K., Hanafi M., Mashohor S., Fazlil iIahi W.F. (2021). Machine learning in medical plants recognition: A review. Artif. Intell. Rev..

[20] Wang A., Zhang W., Wei X. (2019). A review on weed detection using ground-based machine vision and image processing techniques. Comput. Electron. Agric..

[21] Bruni E., Jensen A.P., Pedersen E.S., Angelidaki I. (2010). Anaerobic digestation of maize focusing on variety, harvest time and pretreatment. Appl. Energy.

[22] Schittenhelm S. (2008). Chemical composition and methane yield of maize hybrids with contrasting maturity. Eur. J. Agron..

[23] Gnyp M.L., Miao Y., Yuan F., Ustin S.L., Yu K., Yao Y., Huang S., Bareth G. (2014). Hyperspectral canopy sensing of paddy rice abovground biomass at different growth stages. Field Crop. Res..

[24] Barzin R., Pathak R., Lotfi H., Varco J., Bora G.C. (2020). Use of UAS Multispectral Imagery at Different Physiological Stages for Yield Prediction and Input Resource Optimization in Corn. Remote Sens..

[25] Ji Z., Pan Y., Zhu X., Wang J., Li Q. (2021). Prediction of Crop Yield Using Phenological Information Extracted from Remote sensing Vegetation Index. Sensors.

[26] Abendroth L.J., Elmore R.W., Boyer M.J., Marlay S.K. (2011). Corn Growth and Development.

[27] Žydelis R., Dechmi F., Isla R., Weihermüller L., Lazauskas S. (2021). CERES-Maize model performance under mineral and organic fertilization in nemoral climate conditions. Agron. J..

[28] Žydelis R., Lazauskas S., Povilaitis V. (2018). Biomass accumulation and N status in grain maize as affected by mineral and organic fertlizers in cool climate. J. Plant Nutr..

[29] Qader S.H., Dash J., Atkinson P.M. (2018). Forecasting wheat and barley crop production in arid and semi-arid regions using remotely sensed primary productivity and crop phenology: A case study in Iraq. Sci. Total Environ..

[30] Žydelis R., Weihermüller L., Herbst M., Klosterhalfen A., Lazauskas S. (2018). A model study on the effect of water and cold stress on maize development under nemoral climate. Agric. For. Meteorol..

[31] Kottek M., Grieser J., Beck C., Rudolf B., Rubel F. (2006). World Map of the Köppen-Geiger climate classification updated. Meteorol. Z..

[32] Bukantis A. (2009). Agroclimatic zoning. Lithuanian National Atlas.

[33] Nikitin B.A. (1999). A method for soil humus determination. Agric. Chem..

[34] Matsushita B., Yang W., Chen J., Onda Y., Qiu G. (2007). Sensitivity of the Enhanced Vegetation Index (EVI) and Normalized Di_erence Vegetation Index (NDVI) to Topographic E_ects: A Case Study in High-Density Cypress Forest. Sensors.

[35] Wu W. (2014). The Generalized Di_erence Vegetation Index (GDVI) for Dryland Characterization. Remote Sens..

[36] Gitelson A.A., Merzlyak M.N. (1997). Remote Estimation of Chlorophyll Content in Higher Plant Leaves. Int. J. Remote Sens..

[37] Raper T.B., Varco J.J. (2015). Canopy-Scale Wavelength and Vegetative Index Sensitivities to Cotton Growth Parameters and Nitrogen Status. Precis. Agric..

[38] Rouse J.W., Hass R.H., Schell J.A., Deering D.W., Harlan J.C. (1974). Monitoring the Vernal Advancement and Retrogradation (GreenWave E_Ect) of Natural Vegetation [Great Plains Corridor].

[39] Roujean J.L., Breon F.M. (1995). Estimating PAR Absorbed by Vegetation from Bidirectional Reflectance Measurements. Remote Sens. Environ..

[40] Rondeaux G., Steven M., Baret F. (1996). Optimization of Soil-Adjusted Vegetation Indices. Remote Sens. Environ..

[41] Louhaichi M., Borman M.M., Johnson D.E. (2001). Spatially Located Platform and Aerial Photography for Documentation of Grazing Impacts on Wheat. Geocarto Int..

[42] Woebbecke D.M., Meyer G.E., Von Bargen K., Mortensen D.A. (1995). Coloer Indices for Weed Identification under Various Soil, Residue, and Lighting Conditions. Trans. ASAE.

[43] Bendig J., Yu K., Aasen H., Bolten A., Bennertz S., Broscheit J., Gnyp M.L., Bareth G. (2015). Combining UAV-based plant height from crop surface models, visible, and near infrared vegetation indices for biomass monitoring in barley. Int. J. Appl. Earth Obs. Geoinf..

[44] Verrelst J., Schaepman M.E., Koetz B., Kneubühler M. (2008). Angular sensitivity analysis of vegetation indices derived from CHRIS/PROBA data. Remote Sens. Environ..

[45] Hunt J.R.R., Daughtry C.S.T., Eitel J.U.H., Long D.S. (2011). Remote Sensing Leaf Chlorophyll Content Using a Visible Band Index. Agron. J..

[46] Gitelson A., Kaufman Y.J., Stark R., Rundquist D. (2002). Novel algorithms for remote estimation of vegetation fraction. Remote Sens. Environ..

[47] Akbarian S., Xu C., Wang W., Ginns S., Lim S. (2020). An investigation on the best-fit models for sugarcane biomass estimation by linear mixed-effect modelling on unmanned aerial vehicle-based multispectral images: A case study of Australia. Inf. Process. Agric..

[48] Breiman L. (2001). Random Forests. Mach. Learn..

[49] Brereton R.G., Lloyd G.R. (2010). Support Vector Machines for classification and regression. Analyst.

[50] Wallach D., Wallach D., Makowski D., Jones J.W. (2006). Evaluating crop models. Working with Dynamic Crop Models.

